# CusS-CusR Two-Component System Mediates Tigecycline Resistance in Carbapenem-Resistant *Klebsiella pneumoniae*

**DOI:** 10.3389/fmicb.2019.03159

**Published:** 2020-01-28

**Authors:** Dongjie Chen, Yunan Zhao, Yanqin Qiu, Liying Xiao, Huaqiang He, Dongmei Zheng, Xiaoqin Li, Xiaoli Yu, Nengluan Xu, Xinlan Hu, Falin Chen, Hongru Li, Yusheng Chen

**Affiliations:** ^1^Shengli Clinical Medical College of Fujian Medical University, Fuzhou, China; ^2^Clinical Microbiology Laboratory, Fujian Provincial Hospital, Fuzhou, China; ^3^Department of Pulmonary and Critical Care Medicine, Fujian Provincial Hospital, Fuzhou, China

**Keywords:** *Klebsiella pneumoniae*, carbapenem resistance, tigecycline resistance, CusS-CusR two-component system, RNA sequencing

## Abstract

**Background:**

The increase in carbapenem-resistant *Klebsiella pneumoniae* (CRKP), especially the emergence of tigecycline-resistant *K. pneumoniae* (KP), is a serious public health concern. However, the underlying mechanism of tigecycline resistance is unclear. In this study, we evaluated the role of the CusS-CusR two-component system (TCS), which is associated with copper/silver resistance, in tigecycline resistance in CRKP.

**Methods:**

Following the *in vitro* evolution of tigecycline-resistant KP, the minimum inhibitory concentrations of tigecycline were determined using the micro-broth dilution method. RNA sequencing and data analysis were performed to identify differentially expressed genes. Quantitative PCR (qPCR) was performed to verify the genes of interest. Genes associated with tigecycline resistance, such as *ramR*, *tex (T)*, and *tet (A)*, were detected by PCR, and then mutants were confirmed by sequencing. Additionally, the efflux pump-associated genes *soxS*, *oqxA*, *oqxB*, *acrE*, and *acrF* were also analyzed by qPCR. *CusR* was deleted and complemented by the suicide vector pKO3-Km plasmid and pGEM-T-easy plasmid, respectively.

**Results:**

Nine strains of KP were evaluated in our study. Strains *A2* and *A3* were evolved from *A1*, *B2*, and *B3* were evolved from *B1*, and *C2* and *C3* were evolved from *C1*. The tigecycline minimum inhibitory concentration for *A1*, *B1*, and C1 was 0.5 μg/mL; that for *A2*, *B2*, and *C3* was 16.0 μg/mL; and that for *A3*, *B3*, and *C3* was 32.0 μg/mL. RNA-sequencing and qPCR confirmed that the differentially expressed genes *cusE*, *cusS*, *cusR*, *cusC*, *cusF*, *cusB*, and *cusA* showed higher expression in *C2* and *C3* than in *C1*. Genes related to the efflux pump AcrAB-TolC showed higher expression in *B2* and *B3* than in *B1*. No mutants of *ramR*, *tex (T)*, or *tet (A)* were detected. *SoxS*, *oqxA*, *oqxB*, *acrE*, and *acrF* did not show increased expression in any group. After deletion and complementation of cusR among C3, the MIC of tigecycline decreased to 4 μg/mL, and then recovered to 32 μg/mL. The expression of *cusFBCA*, correspondingly decreased and increased significantly.

**Conclusion:**

In addition to its primary function in resistance to copper/silver, the CusS-CusR two-component system is associated with CRKP resistance to tigecycline.

## Introduction

In recent decades, multiple drug-resistant and carbapenem-resistant Enterobacteriaceae have increased in abundance, particularly *Klebsiella pneumoniae* (*KP*), *Escherichia coli*, *Enterobacterial* spp., *Proteus* spp., and *Serratia mucosa*, greatly limiting the efficacy of antibiotic treatment. Tigecycline is currently widely used in clinical practice as an anti-infective treatment and has become the most important antibiotic for treating carbapenem-resistant *Acinetobacter baumannii* and carbapenem-resistant *Enterobacteriaceae.* However, *A. baumannii*, *E. coli*, and *K. pneumoniae* have shown resistance to tigecycline. Additionally, various mechanisms have been reported to confer tigecycline resistance, with most studies focusing on resistance-nodulation cell division transporters, mainly among AcrAB-TolC efflux pumps, while other pumps and various control pathways are also reported to be associated with tigecycline resistance (Pournaras et al., 2016; Chiu et al., 2017; Ye et al., 2017; He et al., 2018).

Sheng et al. (2014) determined the tigecycline resistance mechanism in 26 strains of tigecycline-non-susceptible KP and found that the resistance of nine strains could not be explained by existing mechanisms. Recent studies revealed a co-regulatory effect between antibiotic resistance and metal resistance. Wang et al. (2017) reported that the CzcR-CzcS two-component system (TCS) in *Pseudomonas aeruginosa* participates in metal detoxification and antibiotic resistance, mainly by co-regulating cross-resistance between Zn (II) and carbapenems antibiotics. Perron et al. (2004) reported that in *P. aeruginosa*, the CzcR-CzcS TCS induced resistance to cadmium, zinc, and cobalt through over-expression of the efflux pump system, resulting in an increase in the MICs of carbapenems. In a study of metal resistance and its relationship with antibiotic resistance, Pal et al. (2017) found that metal resistance and antibiotic resistance genes were located at the same site in the cell, and a single resistance mechanism (such as the efflux pump) could result in resistance to either antibiotics or metal. Hölzel et al. (2012) studied the relationship between heavy metals and bacterial antibiotic resistance in pig manure, and revealed that the presence of copper and zinc was significantly correlated with the increased drug resistance rate of β-lactam antibiotics, while the presence of mercury was significantly correlated with the low drug resistance of *E. coli* to β-lactams, aminoglycosides, and other antibiotics. These studies suggest that bacterial resistance to metals is associated with antibiotic resistance.

**FIGURE 1:**
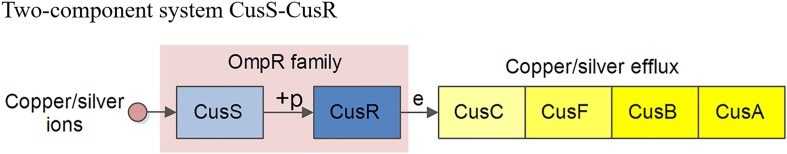
KEGG pathway of the CusS-CusR TCS.

The CusS-CusR TCS is associated with bacteria resistant to the heavy metals copper/silver, in which copper/silver ions stimulate the phosphorylation of the outer membrane protein, CusS; the phosphorylated CusS phosphorylates the translation regulator, CusR. The phosphorylated CusR promotes higher expression of the genes encoding the efflux pump CusCFBA, which pumps the copper/silver out of the bacteria, thus leading to bacteria that are resistant to heavy metals. The Kyoto Encyclopedia of Genes and Genomes (KEGG) pathway of CusS-CusR TCS is shown in Figure 1.

However, only a few studies have focused on the tolerance of *K. pneumoniae* to heavy metals and its association with antibiotic resistance. Accordingly, in this study, we induced three forms of carbapenem-resistant *Klebsiella pneumoniae* (CRKP) resistance to tigecycline *in vitro* and identified the differentially expressed genes using transcriptome sequencing. In particular, we focused on the CusS-CusR TCS to determine its potential role in tigecycline resistance in nine CRKP strains that evolved from the three parental strains *KPN222 (A)*, *KPN114 (B)*, and *KPN315 (C)*.

## Materials and Methods

### Antimicrobial Susceptibility Testing

Antibiotic susceptibility testing was performed using the Vitek 2 system (BioMérieux, Marcy-l Étoile, France). The MICs of tigecycline and polymyxin B were determined using the micro-broth dilution method (BIO-KONT Co., Ltd., Wenzhou, China). The breakpoints of tigecycline and polymyxin B were interpreted according to the Clinical and Laboratory Standards Institute guidelines (Kuti et al., 2018). *E. coli* ATCC25922 and *P. aeruginosa* ATCC27853 were used as quality controls.

### Laboratory Evolution of Tigecycline-Resistant Mutants

Independent single colonies of the CRKP strains *KPN222 (A)*, *KPN114 (B)*, and *KPN315 (C)* were grown overnight at 37°C. The cultures were then inoculated into Luria–Bertani (LB) agar with serially increasing concentrations of tigecycline, starting at a value equal to half of the MIC of the original strain and doubled when bacteria grew on the plates; the final concentration of tigecycline was 256 μg/mL. The resistant strains were transferred to a blood agar plate and continuously transferred 10 times, and then the MIC of tigecycline was determined to obtain the stable resistant strain. The tigecycline resistant strains were stored at −80°C until analysis.

### RNA Extraction, Sequencing, and Data Analysis

The isolates were grown in high-osmolality LB broth at 37°C until the early exponential phase, and then RNA was extracted using the RNApure Bacteria kit (CWBio Co., Beijing, China) according to the manufacturer’s protocol. The concentration of RNA was measured using a Nanodrop spectrophotometer (Thermo Fisher Scientific, Waltham, MA, United States). RNA sequencing was performed using a HiSeq 2000 sequencer (Illumina, San Diego, CA, United States) and *de novo* assembly was carried out using Trinity software^1^. BLASTn (Altschul et al., 1990) was used to analyze Unigene NT with a Blastx (Altschul et al., 1990) or Diamond (Buchfink et al., 2015) search on Unigene for NR; KOG, KEGG, and SwissProt were used to analyze Blast2GO (Conesa et al., 2005) and the NR annotation results for Gene Ontology (GO) annotation. Bowtie 2 (Langmead and Salzberg, 2012) was used to compare the clean reads with the corresponding sequences in Unigene, and RSEM (Li and Dewey, 2011) was then used to calculate the gene expression levels of each sample. The PossionDis method was used for differential gene detection.

### Quantitative PCR and Standard PCR

The expression levels of the CusS-CusR TCS were determined using quantitative qPCR. The primers used for the CusS-CusR TCS and other genes associated with tigecycline resistance are shown in Table 1. The primers used for the AcrAB-TolC efflux pump-associated genes *soxS*, *acrB*, *rarA*, *ramA*, *ramR*, *marA*, *tolC*, and *acrA* were obtained from the literature (He et al., 2015). The primers associated with the SdiA-AcrEF and RarA-OqxAB efflux pump are also shown in Table 2. Total bacterial RNA was extracted using the RNA Pure Bacteria kit (CWBIO Co., Beijing, China). cDNA was then synthesized using a Revert Aid First Strand cDNA Synthesis kit (Thermo Fisher Scientific). Finally, quantitative PCR (qPCR) was performed with the UltraSYBR Mixture kit (CWBIO Co., Beijing, China) using a Cobas z 480 analyzer (LightCycler 480, Roche, Basel, Switzerland) with initial incubation at 95°C for 10 min, followed by 40 cycles of 15 s at 95°C and 60 s at 60°C. The melting curve fluorescence was evaluated at five times per degree Celsius from 60 to 95°C. Each reaction was carried out in triplicate. For all samples, a housekeeping gene (16S rRNA-F: 5′-GTGCCAGCMGCCGCGGTAA-3′, 16S rRNA-R: 5′-GGCGTGGACTTCCAGGGTATCT-3′) (Chen et al., 2007) was used to normalize the expression of genes. The threshold cycle (CT) numbers were confirmed using the detection system software, and the data were analyzed using the 2^ΔΔCt^ method. Gene expression levels were determined and compared with those of wild-type strains (*A1*, *B1*, and *C1*, expression = 1).

**TABLE 1:** Primers of CusS-CusR TCS and other genes associated with tigecycline resistance.

**Genes**	**Primers F/R (5′→3′)**	**Length (nt)**	**Annealing**
			**temperature (°C)**
*cusE*	CGGATTGCCCGTTATTC GGGGACTGCTCCTCGTAT	230	55
*cusS*	GCAACAAATCAGCACCAC GCTAAAGAGCACTTCACCC	148	55
*cusR*	GGCACGATTGAACACAGGG CCGAGAGGTCAGCCACTTT	160	58
*cusC*	TGACAAGCCGACCACACA GCTCAACGAAAGCATAGGAC	257	55
*cusF*	TTGGTGCCTTCTCTGTCA CGTGCGAAATGGTAATCT	162	56
*cusB*	CAGGTCCGTCTCCAGGTTT TTGGTATCAGCAGCATCTCC	103	56
*cusA*	GTGGCGTTGTCAATCTGCT TCCGTTCTGCCCTGGTG	223	57
tex(T)*	GGACCCGTTGGACTGACTA CACCCATTGGTAAGGCTAAG	193	55
tet(A)*	CAGGCAGGTGGATGAGGAA GCAGGCAGAGCAAGTAGAGG	173	58
*acrE*	ATGCCTCCGTGATGTTGG TCCGCTTCCGCTTTGA	175	56
*acrF*	GTGCTCTCTGCGGTATTCG TGAGGCTGGCTTCAACAA	162	55
*oqxA*	CGCAGCTTAACCTCGACTTCA ACACCGTCTTCTGCGAGACC	168	57
*oqxB*	CGAAGAAAGACCTCCCTACC CGCCGCCAATGAGATACA	178	58

*: *tex (T)* and *tet (A)* were detected by standard PCR and confirmed the mutant by Sanger sequencing.

**TABLE 2:** Antibiotics susceptibility of primary strains and induced strains.

**Antibiotics**	**MIC (μg/mL) S/I/R**								
	**A1**	**A2**	**A3**	**B1**	**B2**	**B3**	**C1**	**C2**	**C3**
Amikacin	≤2 S	≤2 S	≤2 S	≤2 S	≤2 S	≤2 S	≤2 S	≤2 S	≤2 S
Tobramycin	≥16 R	≥16R	≥16 R	≥16R	≥16 R	≥16R	4 S	4 S	4 S
Minocycline	≥16 R	≥16R	≥16 R	≥16R	≥16 R	≥16R	≥16 R	≥16R	≥16 R
Trimethoprim–Sulfamethoxazole	≥320 R	≥320 R	≥320 R	≥320 R	≥320 R	≥320 R	≥320 R	≥320 R	≥320 R
Cefoperazone/sulbactam	≥64 R	≥64R	≥64 R	≥64R	≥64 R	≥64R	≥64 R	≥64R	≥64 R
Levofloxacin	≥8 R	≥8 R	≥8 R	≥8 R	≥8 R	≥8 R	≥8 R	≥8 R	≥8 R
Ciprofloxacin	≥4 R	≥4 R	≥4 R	≥4 R	≥4 R	≥4 R	≥4 R	≥4 R	≥4 R
Cefepime	≥32 R	≥32R	≥32 R	≥32R	≥32 R	≥32R	≥32 R	≥32R	≥32 R
Aztreonam	≥64 R	≥64R	≥64 R	≥64R	≥64 R	≥64R	≥64 R	≥64R	≥64 R
Piperacillin–Tazobactam	≥128 R	≥128 R	≥128 R	≥128 R	≥128 R	≥128 R	≥128 R	≥128 R	≥128 R
Ceftazidime	≥64 R	≥64R	≥64 R	≥64R	≥64 R	≥64R	32 R	32 R	32 R
Amoxicillin/clavulanic- acid	≥128 R	≥128 R	≥128 R	≥128 R	≥128 R	≥128 R	≥128 R	≥128 R	≥128 R
Imipenem	≥16 R	≥16R	≥16 R	≥16R	≥16 R	≥16R	≥16 R	≥16R	≥16 R
Meropenem	≥16 R	≥16R	≥16 R	≥16R	≥16 R	≥16R	≥16 R	≥16R	≥16 R
Polymyxin B*	≤0.5 S	≤0.5 S	≤0.5 S	≤0.5 S	≤0.5 S	≤0.5 S	≤0.5 S	≤0.5 S	≤0.5 S
Tigecycline*	0.5 S	16 R	32 R	0.5 S	16 R	32 R	0.5 S	16 S	32 R

MIC, minimal inhibitory concentration; S, sensitive; I, intermediary; R, resistance; *: tigecycline and polymyxin B antibiotic susceptibility were determined by micro-broth dilution method.

The mutations of *tex (T)* and *tex (A)*, which are associated with tetracycline resistance, were detected using the standard PCR. The DreamTaq PCR Master Mix kit (Thermo Fisher Scientific) was used and the reaction system contained 25.0 μL of 2 × Dream Taq PCR Master Mix, 0.4 μL of forward and reverse primers (10 mol/L), and 4.0 μL of DNA template supplemented with 50.0 μL ddH_2_O. The reaction conditions were as follows: 95°C for 1 min; followed by 30 cycles of 95°C at 30 s, 50°C for 30 s, and 72°C for 1 min; with a final extension at 72°C for 8 min. All qPCR and standard PCR products were confirmed using agarose gel electrophoresis and sequencing.

### Deletion and Complementation of the Gene *cusR*

Based on the homologous recombination technology, the deletion strain of the △*cusR* gene was constructed using the suicide vector pKO3-Km plasmid, and the gene fragment containing the activation region and the transcriptional termination region of the coding region of the *C-*△*cusR* gene was amplified. The gene fragment was cloned into the pGEM-T-easy plasmid and transferred to the deletion strain to construct the complemented strain of the *C*-△*cusR* gene. The primers are shown in Table 4.

## Results

### Antibiotic Susceptibility of the Strains

All strains were resistant to tobramycin, minocycline, trimethoprim–sulfamethoxazole, cefoperazone/sulbactam, levofloxacin, ciprofloxacin, cefepime, aztreonam, piperacillin–tazobactam, ceftazidime, amoxicillin/clavulanic acid, imipenem, and meropenem. The strains were only sensitive to amikacin and polymyxin B with MICs of 2 and 0.5 μg/mL, respectively. The tigecycline MIC of *A1*, *B1*, and *C1* was 0.5 μg/mL; that of *A2*, *B2*, and *C2* was 16 μg/mL; and that of *A3*, *B3*, and *C3* was 32 μg/mL. Antibiotic susceptibility of the primary strains and induced strains is shown in Table 2.

### RNA Sequencing

The differentially expressed genes identified by RNA-sequencing are shown in Table 3 and Supplementary Material. Unigene 28_All was 4997 bp long and the NCBI BLAST revealed that it contained *cusE*, *cusS*, *cusR*, *cusC*, and *cusF*. Unigene274_All was 1090 bp long and contained *cusA*. Unigene1733_All was 1111 bp long and contained *cusB*. These three genes showed higher expression in *C2* and *C3* than in *C1*. Unigene1142_All was 2137 bp long, annotated to the transcriptional regulatory gene *ramA* in the genes encoding the AcrAB-TolC efflux pump, and highly expressed in *B2* and *B3*, with *B2/B1* log_2_ fold-change = 3.31 and B3/B1 log_2_ fold-change = 3.61.

**TABLE 3:** Differential expression genes come from RNA sequencing.

**Gene ID**	**log_2_Fold change**	
Unigene 28_All	13.8(*C*2/*C*1)	14.24(*C*3/*C*1)
Unigene274_All	10.59(*C*2/*C*1)	10.94(*C*3/*C*1)
Unigene1733_All	6.32(*C*2/*C*1)	6.52(*C*3/*C*1)
Unigene1142_All	3.31(*B*2/*B*1)	3.61(*B*3/*B*1)

**TABLE 4:** Primers of deletion and complement the gene of *cusR*.

**Name**	**Primer**	**5′→3′**
*△cusR*	C3-△cusR-A	GTATGCGGCCGCCACGACGGGC TTCTTC
	C3-△cusR-B	AGGTCGTCTGGACGGTTTGCATG CTCCCCGGCTGGCTGC
	C3-△cusR-C	GCAGCCAGCCGGGGAGCATGCA AACCGTCCAGACGACCT
	C3-△cusR-D	GTATGCGGCCGCGATTCTCGGAG GTGATGTT
*C-△cusR*	C3-△cusR-F	GAGTCCATGGATGAAAATATTA ATCGTTGA
	C3-△cusR-R	GAGTGTCGACTGGAGATCCCGG ATGCATAG
pKO3-Km	pKO3-Km-F	AATAAGCGGATGAATGGCAG
	pKO3-Km-R	TCCCTCACTTTCTGGCTGG
pGEM-T-easy	pGEM-T-easy-km-F	GCGAATTGGGCCCGACGTC
	pGEM-T-easy-km-R	CGCAGCCGAACGACCGAG
*cusR*	C3-*cusR*-F	GGCACGATTGAACACAGGG
	C3-*cusR*-R	CCGAGAGGTCAGCCACTTT
*16s rRNA*	16s rRNA-F	AAAGCGTGGGGAGCAAACAG
	16s rRNA-R	CCGCTGGCAACAAAGGATAA

*“__” enzyme cutting site; *Nco* I :CCATGG; *Sal* I :GTCGAC; *Not*I :GCGGCCGC.*

### Quantitative PCR

**FIGURE 2:**
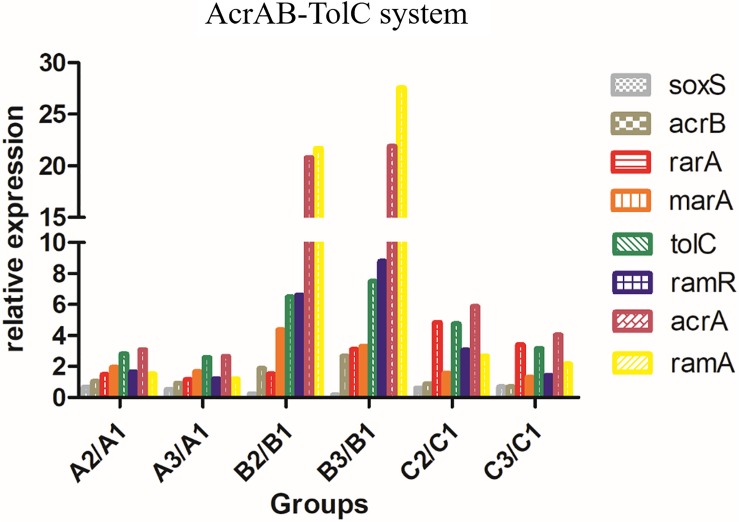
Relative expression of AcrAB-TolC in groups A, B, and C. Efflux pump AcrAB-TolC-associated genes: *soxS*, *acrB*, *rarA*, *ramA*, *ramR*, *marA*, *tolC*, and *acrA*. Group B showed higher expression than groups A and C.

**FIGURE 3:**
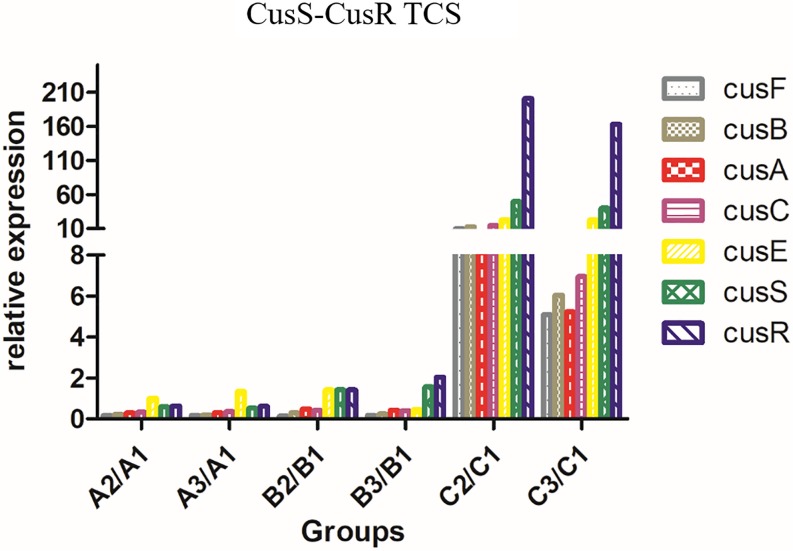
Relative expression of CusS-CusR TCS in groups A, B, and C. CusS-CusR TCS associated genes: *cusF*, *cusB*, *cusC*, *cusA*, *cusS*, *cusR*, and *cusE*. Group C showed higher expression than groups A and B.

The relative expression levels (fold-change) of genes related to the AcrAB-TolC efflux pump (*soxS*, *acrB*, *rarA*, *ramA*, *ramR*, *marA*, *tolC*, and *acrA*) in *A2/A1* and *A3/A1*, *B2/B1* and *B3/B1*, and *C2/C1* and *C3/C1* are shown in Figure 2. The expression level of AcrAB-TolC efflux pump-related factors was the highest in group B. The relative expression levels (fold-change) of genes related to the CusS-CusR TCS (*cusF*, *cusB*, *cusC*, *cusA*, *cusS*, *cusR*, and *cusE*) in *A2/A1* and *A3/A1*, *B2/B1* and *B3/B1*, and *C2/C1* and *C3/C1* are shown in Figure 3. CusS-CusR TCS showed the highest expression in group C.

### Tigecycline Resistance-Associated Genes

We detected the AcrEF-TolC efflux pump and OqxAB-TolC efflux pump in *A2*, *A3*, *B2*, *B3*, *C2*, and *C3*. In contrast, the *acrE*, *acrF*, *oqxA*, and *oqxB* genes did not show higher expression than *A1*, *B1*, and *C1*. The *ramR*, *tex (T)*, and *tet (A)* genes contained no mutations.

### Effect of Deletion and Complementation

Successfully built a non-trace cusS gene deletion strain *△C3* and the complemented strain *C-△C3*, and then verified by RT-PCR of the *cusR* gene in Δ*C3* showing no expression. The tigecycline MIC of *△C3* was decreased to 4 μg/mL and that of *C-△C3* was recovered to 32–4 μg/mL, and the expression of *cusFBCA* correspondingly decreased and increased significantly.

## Discussion

In the present study, we successfully screened three groups of tigecycline-resistant strains by RNA-sequencing, followed by annotation with biological information. We found that the AcrAB-TolC efflux pump system was highly expressed in group B, indicating that this system plays an essential role in the development of CRKP resistance to tigecycline. The AcrAB-TolC efflux pump is involved in resistance-nodulation cell division and has been reported to be resistant to several types of antibiotics (Zhong et al., 2014; Castanheira et al., 2016; Yuhan et al., 2016). However, Nielsen et al. (2014) reported that the IS5 insertion element increased the MIC of tigecycline by four-fold and was not dependent on the functional AcrAB-TolC efflux pump, suggesting that the AcrAB-TolC efflux pump is not the only mechanism causing tigecycline resistance. Hladicz et al. (2017) reported that RamR is a repressor that prevents the overexpression of *ramA*; when *ramR* is mutated, *ramA* is overexpressed, leading to tigecycline resistance; this mechanism was confirmed by Li et al. (2017). However, in the current study, no mutation in *ramR* was detected.

Zhang et al. (2018) reported that impairment of AcrAB-TolC function upregulates *acrEF* quinolone resistance in *E. coli*. Thus, we also detected *acrE* and *acrF*, but no increase in expression was observed. Juan et al. (2016) evaluated 43 strains each of tigecycline-non-susceptible *KP* and tigecycline-susceptible *KP*, and found that the overexpression of OqxAB efflux pumps was a major cause of tigecycline-non-susceptible *KP* resistance to tigecycline. However, the evolved strains in this study did not show an increase in their expression. Furthermore, the genes *tex(T)* and *tet(A)*, which are associated with tigecycline resistance, also did not contain mutations, indicating that these genes were not involved in tigecycline resistance in groups A, B, and C.

Andrade (Andrade et al., 2018) showed that multidrug-resistant *CTX-M*-(15, 9, and 2) – and *KPC-2*-producing *Enterobacter hormaechei* and *Enterobacter asburiae* isolates possessed a set of acquired heavy metal tolerance genes including a chromosomal sil operon (for acquired silver resistance). Yuan et al. (2019) also indicated that mercury/silver resistance genes were associated with antibiotic resistance. Elkrewi et al. (2017) reported that cryptic Ag + resistance pertaining to the sil operon is prevalent and readily activated in particular genera (*Enterobacter* and *Klebsiella*). Conroy et al. (2010) reported that CusCFBA had a narrow substrate spectrum transporting Cu (I) and Ag (I); in this research, the MIC of tigecycline and the expression of c*usCFBA* presented significant changes after deletion and complementation of *cusR*. The c*us* system was clearly elaborated by Pal et al. (2017), however, further research is necessary to verify whether it is involved in cross-resistance or co-resistance to metal and resistance to antibiotics. In conclusion, we considered that in group C, the CusS-CusS TCS was activated and resistance was induced in *C2/C3* strains to tigecycline.

We recognize some limitations to our study. The strains in group A did not show any changes in AcrAB-TolC efflux pump-related and CusS-CusR TCS-related gene expression. Additionally, genes associated with tigecycline resistance such as *acrE*, *acrF*, *oqxA*, and *oqxB* did not show increased expression, and *tex (T)* and *tet (A)* did not contain any mutations. Thus, other mechanisms may have caused *A2* and *A3* resistance to tigecycline, which requires additional analysis.

## Conclusion

Despite these limitations, we found that the increased expression of the CusS-CusR TCS, which is associated with Cu and Ag resistance, mediated CRKP resistance to tigecycline, which may become a novel target of antibiotics. Thus, in cases where the common mechanisms are not identified as mediators of tigecycline resistance, the presence of high CusS-CusR TCS expression may be considered.

## Data Availability Statement

The datasets generated for this study can be found in NCBI SRA https://www.ncbi.nlm.nih.gov/sra/PRJNA596084.

## Author Contributions

YC, DC, and HL designed the experiments, analyzed the data, and wrote the manuscript. YZ, LX, YQ, HH, and DZ performed the experiments and analyzed the data. XL, XY, NX, XH, and FC analyzed the data.

## Conflict of Interest

The authors declare that the research was conducted in the absence of any commercial or financial relationships that could be construed as a potential conflict of interest.

## Footnotes

1 https://github.com/trinityrnaseq/trinityrnaseq/wiki/Output-of-Trinity-Assembly

## Supplementary Material

DATA SHEET S1 Transcriptome sequencing results of groups B and C.
